# Characterization of *GLOD4* in Leydig Cells of Tibetan Sheep during Different Stages of Maturity

**DOI:** 10.3390/genes10100796

**Published:** 2019-10-12

**Authors:** Xia Wang, Taotao Li, Ningbo Liu, Hongyu Zhang, Xingxu Zhao, Youji Ma

**Affiliations:** 1 College of Animal Science and Technology, Gansu Agricultural University, Lanzhou 730070, China; wangx@st.gsau.edu.cn (X.W.); litt@st.gsau.edu.cn (T.L.); liunb@st.gsau.edu.cn (N.L.); zhanghy@st.gsau.edu.cn (H.Z.); 2 Sheep Breeding Biotechnology Engineering Laboratory of Gansu Province, Minqin 733300, China; 3 College of Veterinary Medicine, Gansu Agricultural University, Lanzhou 730070, China; zhaoxx@gsau.edu.cn

**Keywords:** cloning, *GLOD4*, Tibetan sheep, testis, Leydig cells

## Abstract

We have previously reported that glyoxalase domain-containing protein 4 (*GLOD4*) is expressed in sheep testes by proteome analysis, but its roles during testicular development remain unclear. The aim of this study was to understand the expression characteristics and biological functions of the *GLOD4* gene in developmental Tibetan sheep testes. The cDNA sequence of the Tibetan sheep *GLOD4* gene was cloned by the RT-PCR method, and the structural characteristics of the GLOD4 protein were analyzed using relevant bioinformatics software, including ProtParam, TMHMM, Signal P 4.1, SOPMA, and phyre2. The expression patterns and immunolocalization of *GLOD4* were examined in developmental testes derived from three-month-old (3M), one-year-old (1Y), and three-year-old (3Y) Tibetan sheep using quantitative real-time PCR (qRT-PCR), Western blot, immunohistochemistry, and immunofluorescence staining. The sequence analysis showed that the coding sequence (CDS) region of the *GLOD4* gene was 729 bp in length and encoded 242 amino acids. Bioinformatics analysis found that the nucleotide and amino acid sequence of Tibetan sheep *GLOD4* exhibited the highest sequence similarity with goat and chiru, and the least with zig-zag eel, of the species compared. *GLOD4* expressions at both the mRNA and protein levels were significantly higher in the testes of the 1Y and 3Y groups than those in the 3M group (*p* < 0.01). Immunohistochemistry and immunofluorescence results indicated that the GLOD4 protein was mainly localized in the cytoplasm of Leydig cells from Tibetan sheep testes throughout the development stages. These results taken together suggest that the *GLOD4* gene may be implicated in the development of the Leydig cells of Tibetan sheep during different stages of maturity.

## 1. Introduction

The testis is the male gonad, and it has the functions of producing sperm and secreting androgens (such as testosterone). Testosterone is the initiator of spermatogenesis and plays important roles in maintaining the male reproductive function and promoting male secondary sexual characteristics. Spermatogenesis is a complex developmental process that occurs within seminiferous tubules of the testis, where spermatogenic cells undergo a series of changes: Proliferation and differentiation of spermatogonia, meiosis of spermatocytes, and deformation of sperm cells, eventually producing mature sperm [1,2,3]. 

Glyoxalase domain-containing protein 4 (*GLOD4*), a glyoxalase-domain containing protein also known as *HC71*, *CGI-150* or *C17orf25*, belongs to the glyoxalase I family, which was firstly isolated from hepatocellular carcinoma in humans [4]. The gene region of human *GLOD4* is approximately 23 kb in length and comprises 10 exons and nine introns [5]. Moreover, the full-length cDNA sequence of human *GLOD4* is 1814 bp, and it encodes a single open reading frame (ORF) consisting of 313 amino acids [5]. Previous studies document that the *GLOD4* gene is widely expressed in various tissues of insects and humans, and the homologous amino acid sequence of *GLOD4* is even found in Chlamydomonas reinhardtti [4,6,7]. Zhang et al. [8] reported that *GLOD4* may inhibit cell growth through interaction with nudix hydrolase 9 (NUDT9), and this interaction may occur in mitochondria. In addition, experiments in adult male mice have demonstrated that expression of the GLOD4 protein is down-regulated in ciclopirox olamine (CPX) treated embryonic stem cells (ESCs) and multipotent adult germline stem cells (maGSCs), indicating that *GLOD4* affects cell proliferation and differentiation by regulating cell cycle progression [9]. Thus far, reports on the *GLOD4* gene in mammals have been focused on humans [4,5] and mice [10].

Previous works in our laboratory have demonstrated that the GLOD4 protein is differentially expressed in sheep testes at different developmental stages, as analyzed by two-dimensional electrophoresis (2-DE) [11]. The expected full-length coding sequence (CDS) from the sheep *GLOD4* gene is 729 bp (XM_027975163.1). To our knowledge, there are no reports regarding the molecular characterization of the *GLOD4* gene and its potential role during testicular development in sheep, especially Tibetan sheep (*Ovis aries*). Tibetan sheep, as one of the major indigenous small ruminants in China, are distributed mainly in the Qinghai-Tibet Plateau and its adjacent areas, where the environment is harsh, the altitude is high, the temperature is cold, and the air is thin [12,13]. Currently, Tibetan sheep are the most numerous livestock (>50 million) on the Qinghai-Tibet Plateau, providing important livelihoods and income for Tibetan herders [14,15]. Under traditional grazing management, Tibetan sheep mainly rely on natural herbage of local pasture, without feed supplementation, to survive [16,17]. However, the pasture supply has large seasonal fluctuations, and the unbalanced nutrient supply cannot meet the nutrient requirements of Tibetan sheep, which frequently leads to malnutrition, poor growth performance, and poor fertility, thereby seriously affecting the rapid development of Tibetan sheep farming [18,19,20]. Tibetan sheep have late sexual maturity (around one year old), and begin mating at about two years old. This study was therefore conducted to understand the sequence characteristics of the Tibetan sheep *GLOD4* gene and its expression patterns and potential functions during testicular development. Herein, we first cloned the full-length coding sequence of Tibetan sheep *GLOD4* gene using the RT-PCR method. Then, we detected the expression profiles and positive cell distribution of the *GLOD4* gene in Tibetan sheep testes at different development stages. We, for the first time, demonstrated that *GLOD4* may be implicated in the functional maintenance of Leydig cells during sheep spermatogenesis. These results will provide additional clues for further understanding the biological functions and regulatory mechanisms of the *GLOD4* gene in testicular development and spermatogenesis in sheep, and even in other mammals.

## 2. Materials and Methods

### 2.1. Experimental Animals and Design

All animals were managed according to the animal care and experimental procedure guidelines approved by the Animal Committee of Gansu Agricultural University (ethics approval number GSAU-AEW-2017-0003). According to the birth record, 24 purebred male Tibetan sheep from three developmental stages, including pre-puberty (3-month-old, 3M; *n =* 8), sexual maturity (1-year-old, 1Y; *n =* 8) and adult (3-year-old, 3Y; *n =* 8), were provided by the Ganjia Tibetan Sheep Breeding Cooperative (Xiahe, Gansu, China). After the sheep were sacrificed, the duplicated testicular tissues were collected for all rams: One sample was rapidly frozen in liquid nitrogen and then stored at −80 °C for the preparation of total RNA and protein, and the other was fixed with 4% paraformaldehyde for about 48 hours, and then used for making paraffin sections.

### 2.2. Total RNA Extraction and cDNA Synthesis

Total RNA from testicular tissues was extracted according to the instructions of TRlzol Reagent (TransGen Biotech, Beijing, China). The integrity of RNA was measured by 1% agarose gel electrophoresis, and the concentration and quality of RNA were determined using an ultra-micro ultraviolet spectrophotometer (Implen, Germany). Then, cDNA was generated for each 500 ng of RNA sample using the EasyScript One-Step gDNA Removal and cDNA Synthesis SuperMix kit (TransGen Biotech, Beijing, China), according to the manufacturer′s instructions.

### 2.3. Cloning of Sheep GLOD4 Gene

According to the predicted mRNA sequence of the ovine *GLOD4* gene in the National Center for Biotechnology Information (NCBI), primers were designed using Primer Premier 6.0 (Premier Company, Canada) (Table 1). The mixed cDNA samples for testicular tissues derived from all rams were used as a template to amplify the CDS sequence of the *GLOD4* gene. The PCR reaction system (25 μL) was comprised of 1 μL of cDNA, 0.8 μL of forward primer, 0.8 μL of reverse primer, 12.5 μL of 2 × Easy Taq PCR SuperMix (TransGen Biotech, Beijing, China), and 10 μL of ddH2O. Reaction procedures were as follows: 1 cycle of 95 °C for 4 min; 40 cycles of 94 °C for 45 s, 64 °C for 30 s; and 1 cycle of 72 °C for 90 s. The PCR product was separated using 1% agarose gel and purified with an EasyPure Quick Gel Extraction kit (TransGen Biotech, Beijing, China). The purified PCR products were ligated to the pEASY-Blunt vector using the pEASY-Blunt Cloning kit (TransGen Biotech, Beijing, China), and then transformed into Trans1-T1 competent cells (TransGen Biotech, Beijing, China) according to operating instructions. The three independent positive clones were selected and sequenced by the Qingke Biological Company (Xi’an, China).

### 2.4. Bioinformatics Analysis of the GLOD4 Gene

The searches for homologous sequences were performed by the BLAST algorithm (http://blast.ncbi.nlm.nih.gov/Blast.cgi). Multiple sequence alignments were performed using DNAMAN software (Lynnon Biosoft, USA). The basic physicochemical properties of GLOD4 were analyzed by ProtParam software (http://web.expasy.org/protparam/). The protein transmembrane region was predicted by TMHMM software (http://www.cbs.dtu.dk/services/TMHMM/). Protein signal peptides were predicted by Signal P 4.1 software (http://www.cbs.dtu.dk/services/SignalP/). The secondary and tertiary structure of the GLOD4 protein were predicted by SOPMA (https://npsa-prabi.ibcp.fr/cgi-bin/npsa_automat.pl?page=npsa_sopma.html) and phyre2 software (http://www.sbg.bio.ic.ac.uk/phyre2/html/page.cgii?id=index), respectively. The evolutionary history, based on the nucleotide sequences, was inferred by MEGA 7.0 [21] using the neighbor-joining (NJ) method [22]. The percentage of replicate trees in which the associated taxa clustered together in the bootstrap test (1000 replicates) were shown next to the branches [23]. The evolutionary distances were calculated using the Kimura 2-parameter method [24].

### 2.5. qRT-PCR

The primers used for qRT-PCR were designed using Primer Premier 6.0 software (Premier Company, Primer Biosoft, Palo Alto, CA, USA and subsequently synthesized by Xi’an Qingke Biological Company, China. The qRT-PCR reactions were performed in a LightCycler 96 Real-Time system (Roche, Basel, Switzerland). Reaction systems with a final volume of 20 μL consisted of 1 μL of cDNA (150 ng), 10 μL of 2 × Fast qPCR Master Mixture (Dining Biotech, Beijing, China), 0.4 μL of each primer (10 μM) (Table 1), and 8.2 μL of RNase-free ddH_2_O. Reaction procedures were as follows: 1 cycle of pre-denaturation at 94 °C for 2 min; 40 cycles of denaturation at 94 °C for 15 s, and annealing at 60 °C for 30 s. β-actin was used as a housekeeping gene to normalize the expression of *GLOD4* mRNA. All experiments were performed in eight biological replicates with three technical replicates each.

### 2.6. Western Blot

Testicular tissues at different development stages were homogenized and lysed using a radioimmunoprecipitation assay (RIPA) protein extraction kit (Solarbio, Beijing, China) and phenylmethanesulfonyl fluoride (PMSF) (Solarbio, Beijing, China), according to operating instructions. Protein concentrations were quantified using a commercial bicinchoninic acid (BCA) protein assay (Beyotime, Shanghai, China). Protein samples were separated by 12% sodium dodecyl sulfate polyacrylamide gel electrophoresis (SDS-PAGE), and then were transferred onto polyvinylidene difluoride (PVDF) blotting membranes (Beyotime, Shanghai, China). The membranes were blocked with phosphate buffered saline tween-20 (PBST) containing 5% non-fat milk for 2 h at room temperature, and then incubated with either rabbit anti-GLOD4 polyclonal antibody (1:500; Bioss, Beijing, China) or anti-beta-actin polyclonal antibody (1:1000; Bioss, Beijing, China) at 4 °C overnight. After being washed with PBST, the membranes were incubated with goat anti-rabbit IgG/HRP antibody (1:5000; Bioss, Beijing, China) for 2 h at 37 °C. After being washed with PBST, the protein signals were visualized using NcmECL Ultra reagents (New Cell & Molecular Biotech Co. LTD, Suzhou, China) in an X-ray room. This experiment was biologically repeated eight times. Each biological replicate consisted of two technical replicates.

### 2.7. Immunohistochemistry and Immunofluorescence

For immunohistochemistry, the paraffin sections were de-paraffinized, rehydrated, and washed thrice with phosphate-buffered saline (PBS). The antigens in specimens was repair retrieved with sodium citrate buffer solution (PH = 7.4; Solarbio, Beijing, China). Immunohistochemistry staining was carried out using a HistostainTM-Plus kit (Bioss Biotechnology Co., LTD, Beijing, China) according to the instructions. The sections were incubated with rabbit anti-GLOD4 antibody (1:100; Bioss Biotechnology Co., LTD, Beijing, China) overnight at 4 °C in a wet box. The PBS replaced the primary antibody as the negative control. Next, positive signals were visualized by a 3′-dia-minobenzidine (DAB) kit (Bioss Biotechnology Co., LTD, Beijing, China). The representative images were captured using an optical microscope (EX31; SOPTOP, Sunny, Ningbo, China) with MvImage software (Sunny, Ningbo, China).

For immunofluorescence, after routine dewaxing and hydration, antigen retrieval in sections was conducted by microwave heating. Sections were treated with an autofluorescence quencher (Servicebio, Wuhan, China), and closed with 5% (w/v) bovine serum albumin (BSA) for 30 minutes at room temperature. Next, sections were incubated with rabbit anti-GLOD4 antibody (1:200; Bioss Biotechnology Co., LTD, Beijing, China) overnight at 4 °C. After being washed with PBS, sections were incubated with FITC conjugated goat anti-rabbit IgG (1:5000; Servicebio, Wuhan, China) at room temperature for 1 h in the dark. The nuclei were stained with 4, 6-diamidino-2-phenylindole (DAPI). Substitution of PBS for the primary antibody served as the negative control. Finally, the anti-fluorescence quencher was used to seal the sections. Images were observed and obtained using a fluorescence microscope (Nikon, Eclipse C1, Tokyo, Japan). For immunohistochemistry and immunofluorescence experiments, eight independent biological replicates were carried out, and each biological replicate was comprised of two technical replicates.

### 2.8. Image Analysis and Data Statistics

The relative expression of *GLOD4* mRNA was calculated by the 2 ^−∆∆Ct^ method [25]. The band density value of the GLOD4 protein was analyzed by AlphaEaseFC image analysis software (Alpha Innotech, USA). Statistical analyses were performed with one-way ANOVA in SPSS 21.0 software (SPSS, Chicago, USA). All results are expressed as means ± SD; *p* < 0.05 indicates the difference was significant, and *p* < 0.01 indicates that the difference was extremely significant. 

## 3. Results

### 3.1. CDS Sequence Characterization of GLOD4

**Figure 1:**
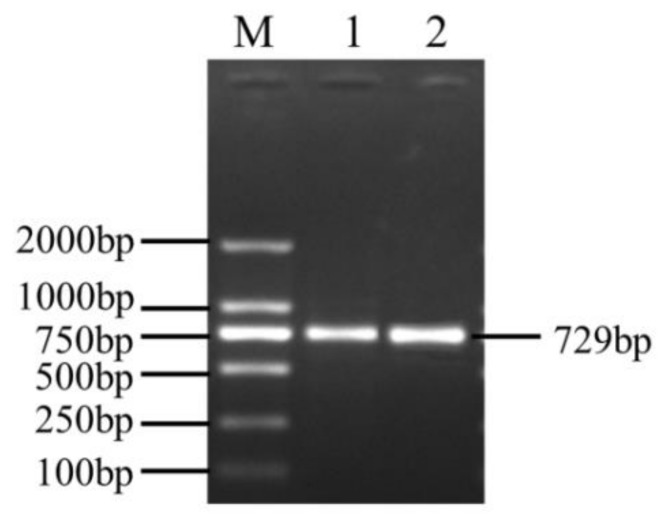
PCR amplification products of the Tibetan sheep GLOD4 coding sequence (CDS) sequence. M, DL 2 000 marker; 1 and 2, PCR product.

**Figure 2:**
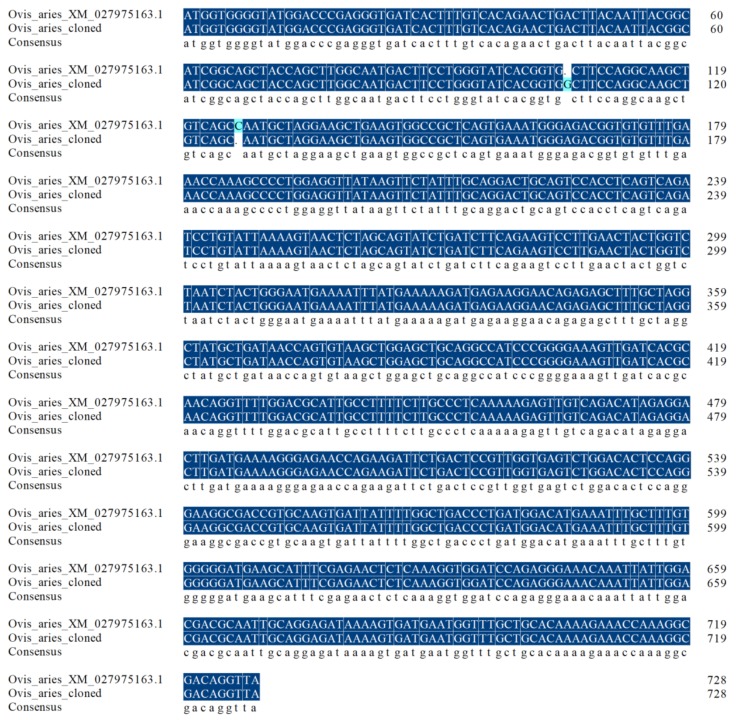
Sequence alignment for predicted and cloned sheep GLOD4 CDS.

**Figure 3:**
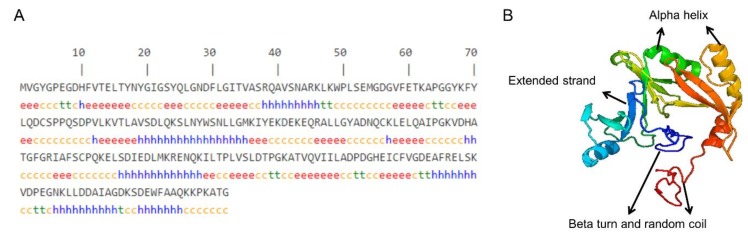
Prediction of structure of the GLOD4 protein. (**A**) Secondary structure of the GLOD4 protein. h (blue), alpha helix; e (red), extended strand; t (green), beta turn; c (yellow), random coil. (**B**) Tertiary structure of the GLOD4 protein.

The PCR amplification product of the *GLOD4* gene was detected and purified by 1% agarose gel electrophoresis. As shown in Figure 1, a specific band of approximately 750 bp indicated that the obtained fragment was within the expected length for use in the experiments. The cloned GLOD4 cDNA sequence of Tibetan sheep included 729 bp, with an ORF encoding translation product of 242 amino acids (accession no.: MN172417; Supplementary Table S1). The cloned nucleotide sequences of GLOD4 were aligned with the predicted sequences from sheep (GenBank no.: XM_027975163.1) in NCBI using BLAST. For the cloned sequence, a G insertion at position 106 and a C deletion at position 127 occurred which were different to the predicted sequence (Figure 2). As analyzed by ProtParam software, the molecular weight, theoretical isoelectric point (pI), and molecular formula of the protein encoded by the *GLOD4* gene was 26654.19D, 4.94, and C_1190_H_1860_N_310_O_368_S_8_, respectively. There were 36 negatively charged amino acid residues (Asp + Glu) and 27 positively charged amino acid residues (Arg + Lys). Sheep GLOD4 protein has a half-life of 30 h in mammalian reticulocytes. The lipid solubility coefficient, average hydrophilicity and instability index for the GLOD4 protein were 82.23, −0.446 and 23.65, respectively. We therefore concluded that the GLOD4 protein of sheep was a hydrophilic stable protein. The results predicted by the TMHMM and Signal P 4.1 software showed that there was no transmembrane region and signal peptide of the GLOD4 protein (Supplementary Figure S1). The predicted secondary structure showed that the GLOD4 protein consisted of 28 alpha-helices (28.1%), 15 beta-turns (6.2%), 91 random coils (37.6%), and 68 extended strands (28.1%) (Figure 3A). The tertiary structures of the GLOD4 protein further spatially extended based on random coils, apha helixes, and beta turns (Figure 3B).

### 3.2. Homology Analysis and Evolutionary Relationships of the GLOD4 Gene among Different Species

**Figure 4:**
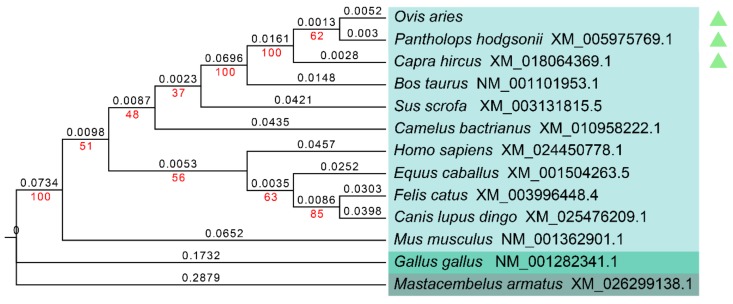
Evolutionary relationships of the *GLOD4* gene among different species. The optimal tree with the sum of branch length = 1.20380259 is shown. The bootstrap values (red) and branch lengths (black) are shown next to the branches. The green arrow indicates that the sheep *GLOD4* gene has the closest homology to chiru (*Pantholops hodgsonii*) and goat (*Capra hircus*).

The nucleotide and amino acid sequences of the Tibetan sheep GLOD4 gene were compared with other species, including closely and more distantly related species. The homology analysis indicated that sheep GLOD4 exhibited the highest similarity with goat and chiru, while showing the lowest similarity with zig-zag eel. The homology of the Tibetan sheep *GLOD4* gene in mammals is above 83% (Table 2). To further understand the evolutionary relationships of the *GLOD4* gene, the phylogenetic trees were then constructed for GLOD4 from different species (Figure 4), and the results displayed that Tibetan sheep *GLOD4* clustered primarily with chiru and clustered posteriorly with goats. It then clustered with mammals such as cattle, pigs, Bactrian camels, horses, cats, dingoes, humans and house mice into a large branch, and finally clustered with the two branches of chickens and zig-zag eels.

### 3.3. Expression Patterns of the GLOD4 Transcript at Different Developmental Stages of Tibetan Sheep Testes

**Figure 5:**
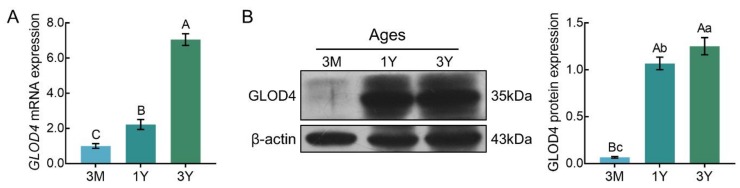
The relative expression levels of GLOD4 in sheep testes at different ages. (**A**) Relative GLOD4 mRNA expression. The bars represent the mean values ± SD from eight independent biological replicates with three technical replicates each. (**B**) Relative GLOD4 protein expression. β-actin was used as a reference gene. The bars represent the mean values ± SD from eight independent biological replicates with two technical replicates each. Different capital letters denote extremely significant differences between groups (*p* < 0.01), while different lower letters denote significant differences between groups (*p* < 0.05). 3M: 3 months old; 1Y: 1 year old; 3Y: 3 years old.

qRT-PCR results showed that *GLOD4* mRNA was expressed in all three stages of testicular tissues, and its expression increased gradually with advancing age (Figure 5A). Similar expression patterns were observed at the protein level, as detected by Western blot. The lowest level of GLOD4 protein was observed in the 3M group, and it was significantly up-regulated in the 1Y group and 3Y group (*p* < 0.01) (Figure 5B).

### 3.4. Localization of the GLOD4 Protein in Developmental Sheep Testes

**Figure 6:**
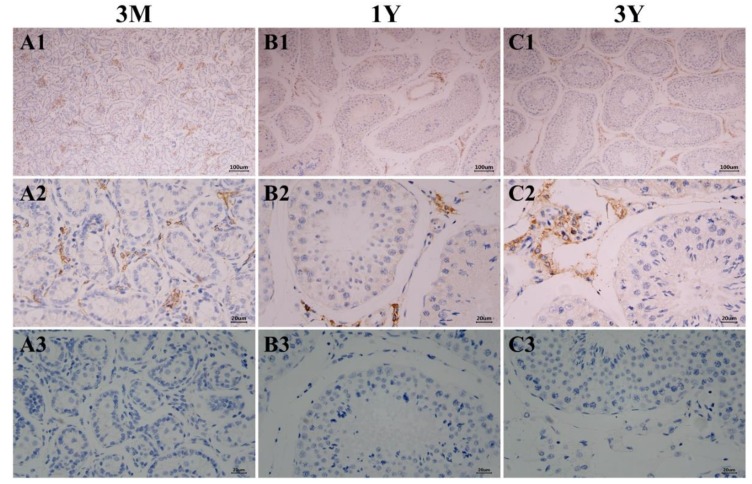
Immunohistochemical staining of the GLOD4 protein in developmental Tibetan sheep testes. (**A1**–**C1**) Immunostaining patterns of GLOD4 protein (brown) in 3M, 1Y and 3Y sheep testes (100×); (**A2**–**C2**) Immunostaining patterns of GLOD4 protein (brown) in 3M, 1Y and 3Y sheep testes (400×); (**A3**–**C3**) The primary antibody was replaced with phosphate-buffered saline (PBS) as the negative controls (400×). The experiment was biologically repeated eight times, and technically repeated two times for each biological replicate. 3M: 3 months old; 1Y: 1 year old; 3Y: 3 years old.

**Figure 7:**
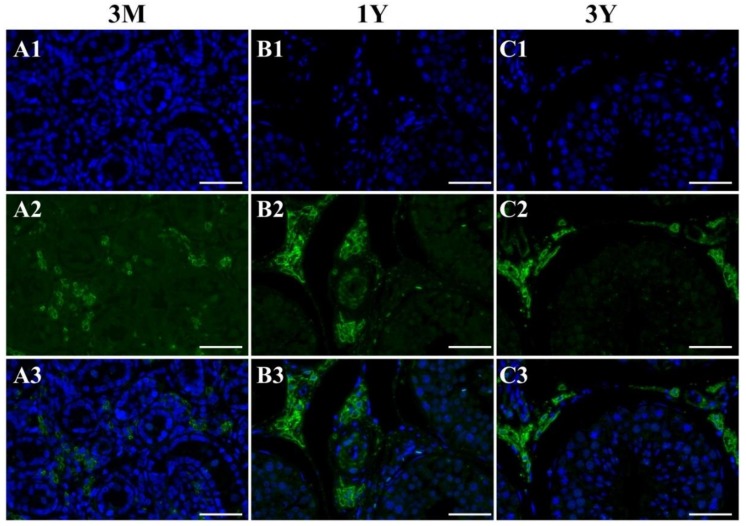
Immunofluorescence staining of the GLOD4 protein in developmental Tibetan sheep testes. (**A1**–**C1**) 4, 6-diamidino-2-phenylindole (DAPI) for nuclei staining (blue); (**A2**–**C2**) GLOD4 protein (green); (**A3**–**C3**) merge; bar = 50 μm. The experiment was biologically repeated eight times, and technically repeated two times for each biological replicate. 3M: 3 months old; 1Y: 1 year old; 3Y: 3 years old.

Examination of positive signals for the GLOD4 protein was performed in sheep testes at different developmental stages by immunohistochemical staining. The results showed that the GLOD4 protein was found in all ages of Tibetan sheep testes and its immunopositive signals were exclusively localized in Leydig cells (Figure 6). Immunofluorescence staining was further performed to explore the subcellular localization of the GLOD4 protein, and the results indicated that the GLOD4 protein was mainly localized in the cytoplasm of Leydig cells from Tibetan sheep testes throughout the developmental stages (Figure 7).

## 4. Discussion

In this work, we cloned the CDS sequence of GLOD4 in Tibetan sheep for the first time by way of the RT-PCR method. The results indicated that the full-length CDS sequence of *GLOD4* was 729 bp, which encoded a total of 242 amino acids. Among them, leucine and lysine account for the largest proportion. Leucine and lysine are essential amino acids in humans and other vertebrates, and play important roles in growth and development, protein synthesis, and control of gene expression through metabolism [26]. It is generally believed that the half-life of a protein is positively correlated with its stability [27]. In this study, the GLOD4 protein of sheep was found to have a half-life of 30 h in mammalian reticulocytes and a low instability index (23.65), indicating that it is stable. Moreover, the alpha helix proportion was as high as 28.1% in the sheep GLOD4 protein structure. Abundant alpha helixes can increase the stability of the protein [28]. These results indicate that the biological function of GLOD4 is stable, which is consistent with previous reports [4]. Sequence homology and phylogenetic tree analysis of *GLOD4* between different species showed that the *GLOD4* gene has high homology among mammals, indicating that *GLOD4* is highly conserved during the evolution of species. 

It has been reported that GLOD4, a mitochondrial protein, is expressed in the liver and kidneys of Blmh^−/−^ mice, but not in wild-type Blmh^+/+^ animals, while *GLOD4* mRNA is expressed in the liver and kidneys of two genotypes, which indicates that the expression of the GLOD4 protein is also regulated post-transcriptionally in Blmh^+/+^ mice and that the regulation is lost in Blmh^−/−^ animals [10,29]. Previous studies have documented that the lack of *GLOD4* and spermatogenesis associated 4 (*SPATA4*) gene can arrest cell cycle progression, and have reported that the genes involved in cilia development also play a role in cell cycle progression [7]. *SPATA4*, a testis-specific gene, is involved in regulating cell proliferation, differentiation, and apoptosis during spermatogenesis [30,31,32]. However, there are few reports on the sheep *GLOD4* gene, and its function is not fully understood. To explore the role of the *GLOD4* gene in sheep testes, we firstly used qRT-PCR and Western blot to detect the expression patterns at both the transcript and protein levels of *GLOD4* in different developmental stages of Tibetan sheep testes. The results showed that the *GLOD4* mRNA and protein were expressed in testes of different ages, and expression trends were similar. The lowest expression of GLOD4 was observed in testes of three-month-old sheep, followed by significant up-regulation in testes from one-year-old and three-year-old Tibetan sheep. These results indicate that GLOD4 may play a vital role in post-puberty Tibetan sheep testes. In order to further understand the biological function of the *GLOD4* gene in Tibetan sheep testes, we examined the distribution of the GLOD4 protein in sheep testes at different developmental stages by using immunohistochemistry and immunofluorescence. The results showed that the GLOD4 protein was mainly detectable in the cytoplasm of Leydig cells. 

For males, the major function of testicular Leydig cells is to produce the male hormone testosterone under the influence of luteinizing hormone (LH). The biosynthesis of testosterone gradually increases with age, and reaches a peak before and after sexual maturity [33]. Significant up-regulation of GLOD4 expression in sheep testes after sexual maturity in this experiment may be due to the increased ability of Leydig cells to synthesize testosterone. LH from the pituitary gland binding to Leydig cell LH receptors stimulates cAMP production [34]. The elevation of cAMP regulates the activation of PKA to stimulate signaling in downstream steroidogenic proteins, increasing the rate of cholesterol translocation into the mitochondria [35]. The first and rate-limiting step in steroidogenesis is the transfer of cholesterol across the inner membrane space from the outer mitochondrial membrane to the inner mitochondrial membrane, a process dependent on the action of steroidogenic acute regulatory (StAR) protein [36,37]. StAR can facilitate the transfer of cholesterol into the mitochondrial matrix and then cytochrome P450scc oxidase (also referred to as CYP11A), located on the inner mitochondrial membrane, can convert it to pregnenolone. Pregnenolone diffuses to the smooth endoplasmic reticulum where it is further metabolized to produce testosterone [38,39]. It has been reported that GLOD4 is a glyoxalase protein that is part of the enzymatic detoxification system in mitochondria, and is involved in mitochondrial function [40]. Studies have found that mitochondria play a key role in steroid synthesis [41,42]. Mitochondrial delta psi (m), ATP synthesis, delta pH and [Ca2+] mt are essential for mitochondrial steroid production [43,44]. Hence, we conclude by speculating that the *GLOD4* gene may play an important role in the mitochondria function of Leydig cells derived from Tibetan sheep testes to regulate the secretion of testosterone, but the exact regulatory mechanism needs to be further verified.

## 5. Conclusions

In summary, this is the first report investigating the CDS sequence characteristics of the *GLOD4* gene, along with first exploring its expression patterns and molecular function, in sheep testes. The CDS region of the sheep *GLOD4* gene was 729 bp in length and encodes 242 amino acids, and the nucleotide sequence is highly homologous to those of other mammalian species. The *GLOD4* mRNA and protein were expressed at lower levels in pre-pubertal testes, however, the expressions were significantly up-regulated in post-pubertal testes. In addition, the GLOD4 protein was localized in the cytoplasm of Leydig cells. These results suggest that the *GLOD4* gene may play an important role in the development of Leydig cells during sheep spermatogenesis.

## Figures and Tables

**Table 1:** The designed primers.

Gene	GenBank No.	Sequence(5′-3′)	Length/bp	Utilization
*GLOD4*	XM_027975163.1	F: ATGGTGGGGTATGGACCCG	729	Cloning
		R: TTAACCTGTCGCCTTTGGTTTC		
*GLOD4*	XM_027975163.1	F: CTCCGTTGGTGAGTCTGG	167	qRT- PCR
		R: ATCTCCTGCAATTGCGTCG		
*β-actin*	NM_001009784.1	F: CTTCCAGCCTTCCTTCCTGG	180	qRT- PCR
		R: GCCAGGGCAGTGATCTCTTT		

**Table 2:** Alignment of the similarity of the GLOD4 nucleotide and amino acid sequence of sheep and other species.

Species	Genbank No.	Nucleotide Similarity %	Amino acid Similarity %
*Pantholops hodgsonii* (chiru)	XM_005975769.1	99.18	100
*Capra hircus* (goat)	XM_018064369.1	99.18	100
*Bos Taurus* (cattle)	NM_001101953.1	96.57	97.52
*Equus caballus* (horse)	XM_001504263.5	87.79	85.95
*Sus scrofa* (pig)	XM_003131815.5	87.52	85.54
*Camelus bactrianus* (Bactrian camel)	XM_010958222.1	87.24	84.71
*Felis catus* (domestic cat)	XM_003996448.4	86.15	85.95
*Canis lupus dingo* (dingo)	XM_025476209.1	85.95	83.88
*Homo sapiens* (human)	XM_024450778.1	85.23	84.3
*Mus musculus* (house mouse)	NM_001362901.1	84.72	83.47
*Gallus gallus* (chicken)	NM_001282341.1	74.54	76.76
*Mastacembelus armatus* (zig-zag eel)	XM_026299138.1	69.74	68.18

## Supplementary Materials

The following are available online at https://www.mdpi.com/2073-4425/10/10/796/s1, Figure S1: Results predicted by the TMHMM and Signal P 4.1 software, Table S1: The analysis of amino acid composition of Tibetan sheep GLOD4.
